# The image and data archive at the laboratory of neuro imaging

**DOI:** 10.3389/fninf.2023.1173623

**Published:** 2023-04-26

**Authors:** Scott C. Neu, Karen L. Crawford, Arthur W. Toga

**Affiliations:** Laboratory of Neuro Imaging, Department of Neurology, USC Mark and Mary Stevens Neuroimaging and Informatics Institute, University of Southern California, Los Angeles, CA, United States

**Keywords:** IDA, data repository, data sharing, data archive, neuroimaging

## Abstract

The Image and Data Archive (IDA) is a secure online resource for archiving, exploring, and sharing neuroscience data run by the Laboratory of Neuro Imaging (LONI). The laboratory first started managing neuroimaging data for multi-centered research studies in the late 1990’s and since has become a nexus for many multi-site collaborations. By providing management and informatics tools and resources for de-identifying, integrating, searching, visualizing, and sharing a diverse range of neuroscience data, study investigators maintain complete control over data stored in the IDA while benefiting from a robust and reliable infrastructure that protects and preserves research data to maximize data collection investment.

## 1. Introduction

The IDA (Toga et al., 2010; Toga and Crawford, 2015; Crawford et al., 2016) is a global resource for storing and disseminating neuroimaging, clinical, biospecimen, and genetic data for national and international consortia efforts (Redolfi et al., 2022) as well as smaller, single-center studies. Locally developed and managed at LONI, clinical data, imaging data, and analysis results are uploaded to the IDA daily, allowing users to obtain data from multiple studies within a single system. This manuscript summarizes recent improvements and developments within the IDA since our last report (Crawford et al., 2016).

Widespread data sharing is supported by IDA web pages that allow study-designated reviewers to receive, evaluate, and approve/disapprove online data use applications. Studies may define preset collections of data that meet specified criteria so that multiple users can access the same sets of data without first needing to conduct searches of the database. Since the IDA keeps extensive records of download activity, users can avoid downloading the same data twice and can easily locate new data after it arrives. There is no requirement to acknowledge the IDA in publications that use data obtained through the IDA, however, study-designated publication policies presented during data use application may specify acknowledgment requirements.

Image and Data Archive data ownership and access policies are defined so that the data belongs solely to its owners and that all data access decisions remain under their direct control. This functionality is often needed by study managers to control access to the data that is being pooled from multiple sites. Permissions to edit and delete data may also be assigned as needed to support review, tracking, and other data management operations. Quality assessments may be conducted by study owners, independent quality control contractors, or by LONI personnel on newly uploaded neuroimaging files that are hidden from users until quality ratings have been assigned.

## 2. Research studies

**FIGURE 1:**
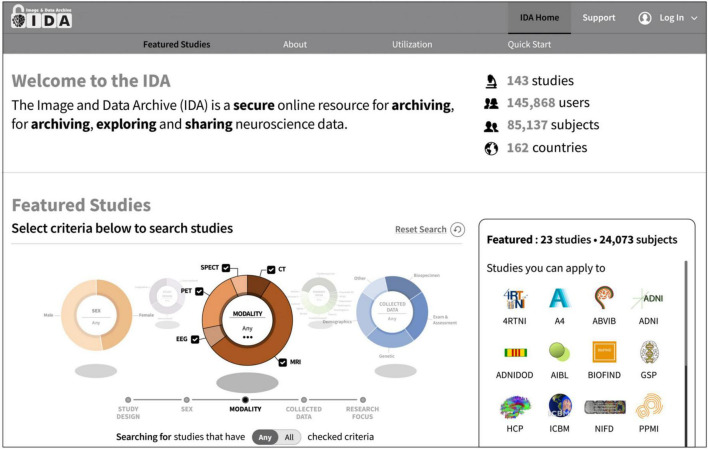
The IDA manages more than 85,000 subjects from over 140 research studies and 270 institutions and receives new data daily. Users search for and apply to featured studies on the IDA home page by searching study criteria such as study design and research focus.

The IDA currently manages data on over 85,000 subjects from more than 140 research studies and 270 institutions and receives new data daily (Figure 1). These studies focus primarily on neurological diseases and conditions, and data has been collected in many different research areas including Alzheimer’s disease, Epilepsy, Parkinson’s disease, and traumatic brain injury (Table 1). While many studies use the IDA exclusively to archive and share data, there are a few studies that mirror data available in other repositories. Each study may provide a logo, set of colors, and a link to an external website that are used to alter the style of IDA web pages. This allows study coordinators to effectively brand the look and feel of the IDA to match each study’s identity.

**TABLE 1:** Representative research studies utilizing the IDA.

Research focus	Study	Study	Institutions	Subjects	Deposit activity	Archived (GB)
Aging	FCDNA	Finance, cognition and default network in aging	1	76	2021–present	325
Aging	HABLE	Health and aging brain among Latino elders study	1	3147	2017–present	3,763
Aging	SLS	Seattle longitudinal study	1	5016	2019–present	113
Alzheimer’s disease/dementia	ADSP_NACC	ADSP Phenotype harmonization consortium	1	3431	2022–present	148
Alzheimer’s disease/dementia	ADPC	Alzheimer’s Disease in primary care	1	483	2019–present	363
Alzheimer’s disease/dementia	ADNI	Alzheimer’s Disease neuroimaging initiative	71	5123	2005–present	7,135
Alzheimer’s disease/dementia	A4	Anti-amyloid treatment in asymptomatic Alzheimer’s	68	4486	2019–2021	790
Alzheimer’s disease/dementia	VCSGT	Biomarkers of ABCA1 mediated functions in Alzheimer’s	1	51	2017–present	259
Alzheimer’s disease/dementia	DHA2BRP	DHA delivery to brain pilot study	2	460	2016–present	916
Alzheimer’s disease/dementia	DVCID	Diverse VCID	9	239	2022–present	256
Alzheimer’s disease/dementia	EEAJ	Estudio de la enfermedad de Alzheimer en Jalisciences	2	181	2016–present	638
Alzheimer’s disease/dementia	GS1	Generation study 1	432	435	2022–present	501
Alzheimer’s disease/dementia	GS2	Generation study 2	927	2446	2022–present	1,438
Alzheimer’s disease/dementia	IDEASHOLD	Imaging dementia–evidence for amyloid scanning	343	10774	2021–present	97
Alzheimer’s disease/dementia	LEADS	Longitudinal early-onset Alzheimer’s disease study	18	576	2018–present	1,440
Alzheimer’s disease/dementia	DVR	Model-based cerebrovascular markers for diagnosing MCI or AD	3	168	2019–present	115
Alzheimer’s disease/dementia	SCAN_AL	SCAN legacy	4	420	2021–present	90
Alzheimer’s disease/dementia	SCAN	Standardized centralized Alzheimer’s neuroimaging	29	2225	2021–present	916
Alzheimer’s disease/dementia	VCID	Vascular cognitive impairment and dementia	1	205	2017–present	307
Alzheimer’s disease/dementia	VCD	Vascular cohort study	2	186	2015–present	699
Cerebrovascular disease	CHBC	Cardiovascular and HIV/AIDS effects on brain and cognition	4	520	2009–present	835
Cerebrovascular disease	PPG	Vascular contributions to dementia and genetic risk factors	3	452	2016–present	730
COVID-19	CVB	COVID-BRAIN	5	53	2021–present	138
Down syndrome	ABCDSU19	Alzheimer biomarker consortium–down syndrome	8	139	2021–present	196
Down syndrome	ADDS	Biomarkers of AD in adults with down syndrome	1	149	2019–present	436
Down syndrome	NIAD	Neurodegeneration in aging Down syndrome	6	250	2016–present	237
Epilepsy	EPIBIOS4	Epilepsy bioinformatics study for antiepileptogenic therapy	17	307	2016–present	2,221
Frontotemporal lobar degeneration	ALLFTD	ARTFL LEFFTDS longitudinal frontotemporal lobar degeneration	23	892	2020–present	1,258
Frontotemporal lobar degeneration	4RTNI	Four repeat Tauopathy neuroimaging initiative	5	129	2011–2016	147
Frontotemporal lobar degeneration	4RTNI2	Four repeat Tauopathy neuroimaging initiative cycle 2	8	257	2017–present	210
Frontotemporal lobar degeneration	LEFFTDS	Longitudinal evaluation of familial frontotemporal dementia	18	909	2015–2020	588
Lifestyle intervention (Alzheimer’s)	GEMS	Gene, exercise, memory and neurodegeneration in blacks study	2	143	2010–present	49
Lifestyle intervention (Alzheimer’s)	LA_FINGERS	LatAm-FINGERS	10	381	2022–present	178
Lifestyle intervention (Alzheimer’s)	POINTER	POINTER Imaging	6	809	2020–present	495
Parkinson’s disease	BIOFIND	BioFIND	8	232	2012–present	1
Parkinson’s disease	DODPD	DOD US army PD cognition longitudinal study	2	38	2018–2021	137
Parkinson’s disease	PPMI	Parkinson’s progression markers initiative	50	4314	2010–present	2,936
Stroke	SPAN	Stroke preclinical assessment network	7	2981	2020–present	2,117
Traumatic brain injury	ADNIDOD	Effects of TBI and PTSD on Alzheimer’s disease in Vietnam vets	19	463	2013–2020	744
Traumatic brain injury	TRACKTBI	Transforming research and clinical knowledge in TBI	18	3569	2014–2022	3296

The total amount of neuroimaging files archived for each study is given in gigabytes (GB).

**FIGURE 2:**
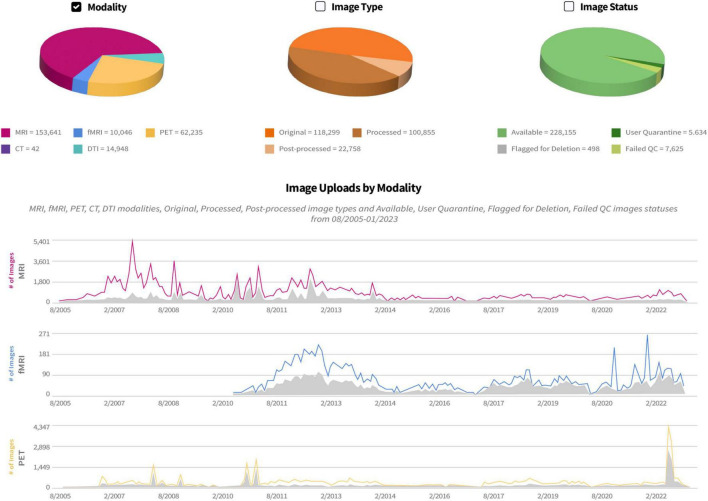
IDA study management pages provide a history of uploads and downloads of each neuroimaging modality. Counts of image uploads are categorized as original (images archived directly from scanner), processed (images corrected for artifacts, etc.) quarantined (removed from general view), and failed (not passing quality control).

Originally conceived and developed as an image archive for magnetic resonance imaging (MRI) files, to date the IDA continues to collect neuroimaging scans from multiple modalities (Figure 2). Currently the IDA manages approximately 90% MRI [73% structural, 10% functional, and 17% diffusion tensor imaging (DTI)], 5% positron emission tomography (PET), and 5% other modalities such as computed tomography (CT), single-photon emission computed tomography (SPECT), and electroencephalogram (EEG). Additionally, the IDA stores files from other types of data, including clinical, electronic patient-reported outcomes (ePRO), proteomics, genetic (DNA/RNA), biospecimen analysis [cerebrospinal fluid (CSF), Fibroblast, peripheral blood mononuclear cells (PBMC), plasma, serum, urine, whole blood, cell line/induced pluripotent stem cells (iPSCs)], digital sensor (smart watch/smart phone), metabolomics, and proteomics.

## 3. Uploading and de-identifying data

The IDA website invites investigators to contact us via email to learn more about using the repository for their study. A welcome package with information about the repository and a form for gathering study details is provided to interested study contacts. Study investigators are asked to complete the form and submit study protocol and informed consent documents for USC IRB review. The completed form is used to assess whether the IDA is a good fit for the study’s data, to determine the scope of work needed, and to estimate costs. A DTA/DUA template is available but local DTAs/DUAs can be accepted with USC Compliance Office approval.

Neuroimaging data files are de-identified and uploaded to the IDA using an executable Java jar file that implements the Java FX framework. This provides a graphical user interface (GUI) that guides users through the de-identification and upload steps. For easy installation, separate installers are available and have been code-signed for the Windows, Mac, and Linux operating systems, and each installer provides its own copy of the Java 15 runtime environment. Many neuroimaging data file formats are supported, including ANALYZE, DICOM, ECAT, EDF, FDF, FreeSurfer, GE, INTERFILE, MINC, NIFTI, NRRD, multi-image TIFF files (in regular and “big” TIFF format) and selected variants of MP4 video files. Target files are read and de-identified at each local institution before the de-identified files are sent to the IDA for archiving. Unlike previous IDA uploaders, the current uploader does not require a temporary working directory. The uploader is also self-updating; after installation the latest updates are automatically retrieved each time the uploader is started. After a user logs in, selects an IDA study, and specifies the user’s site, the user enters a replacement subject ID and the directory path of files to upload. The file format of each file is automatically identified, and the appropriate de-identification removes patient-identifying information. De-identifications can be customized for the needs of each study, but in general all de-identifications replace the patient’s name and ID fields with the user-supplied research identifier, remove all fields that are non-numeric unless otherwise specified, and replace unique identifier fields with hashed values. If required, obfuscation of binary content (e.g., images) is performed by image experts before uploading. A progress bar displays the total number of files that have been de-identified and uploaded to the IDA server along with reports of any files that have been rejected using de-identification criteria specific to each study. After all files have been uploaded, the user is directed to an IDA web page where additional study-specific information is entered, and the upload is finished. Copies of all files archived in the IDA are backed up in the cloud using the Amazon AWS S3 Glacier service.

More advanced users invoke a command line version of the Java uploader to perform batch uploads. This batch process requires a CSV file that must contain at least two columns; one column for the replacement subject ID and one column for the path of the files to upload. Each row of the CSV file identifies a separate upload. The batch uploader processes each row of the CSV file and writes a new CSV file as output. This new progress CSV file contains all the information provided in the first CSV file with additional columns describing the ID assigned to the upload, the LONI UID created for the uploaded files, the numbers of uploaded files and their file formats, and a column that provides the status of each upload. If a study requires additional information for an upload, new empty columns are also created. After users enter missing information and correct any status errors reported in the progress CSV file, they run the command line uploader again with the progress CSV file as input. Additional progress files are created until all uploads have completed. At the end of the batch upload process, the last progress CSV file provides users with a receipt containing detailed information about each upload.

For all neuroimaging data uploads, newly uploaded files are checked against previously uploaded files for duplicates using hashes and image header fields. When a duplicate is detected, the newly uploaded file replaces the previously uploaded file. In practice, many uploaders find duplicate detection and removal essential since in general institutions tend to focus more on image acquisition and less on local data management. As most uploads are files copied directly from imaging scanners, we have not encountered sufficient need to support multiple versions of the same upload. Optionally, non-duplicate neuroimaging data may be placed into one or more download queues for study personnel who wish to receive all newly uploaded data. They typically invoke an IDA download queue API on a nightly basis to get all data uploaded to a study for each day. Users can locate newly uploaded data by searching the IDA for all neuroimaging data they have not yet downloaded. Often after a study begins and de-identified neuroimaging data files have been archived in the IDA, study coordinators make requests for additional image header metadata changes. For example, new subject ID substitutions or higher levels of de-identification may be required. One common request is to retroactively “shift” all dates so that the time difference between any two dates in every neuroimaging data file is unchanged for the same subject. This involves selecting a date difference for each subject and replacing all neuroimaging image data files with the date-shifted metadata. This post-processing of archived neuroimaging files can be automatically applied to new image uploads or retroactively to all archived files in an IDA study.

## 4. Data management

**FIGURE 3:**
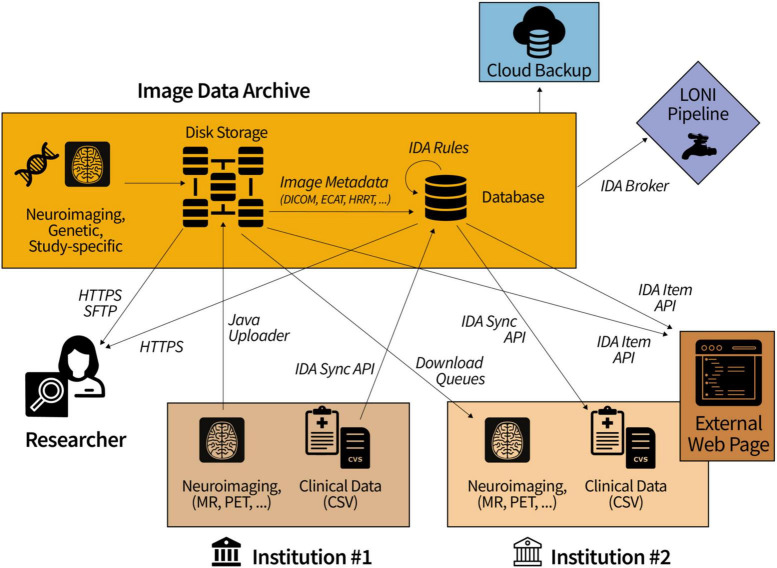
Collaborating institutions transfer data to and from the IDA while study committees allow access to approved researchers. In the diagram, Institution #1 uploads locally-acquired neuroimaging scans and clinical data. Download queues and the IDA Sync API make this data readily accessible to other institutions (e.g., Institution #2) for processing and reuploading.

Since the IDA functions as a hub for data transfers between collaborating groups (Figure 3), many studies require tabular data uploaded to the IDA to be processed and/or combined with other data before it becomes available for downloading. Data harmonization, quality control processing, and/or further de-identification is conducted in about one half of all studies featured on the IDA home page. To support these data mapping aims, we have developed an SQL-like language to create, edit, and execute transformations on database tables. A Java client provides a command line interface to add and remove “rules” that are executed by the client on data stored in the IDA database. Built on top of MySQL commands, IDA rule commands provide extra functionality to create and execute loops as well as to define variables. Statements in an IDA rule script are indented with white space similar to the Python programming language and are imported and exported from the IDA using the Java client. These rules can be executed manually, as part of cron jobs, or can be triggered after tabular data is uploaded to the IDA. Each line of a rule script may be associated with a variable that represents all output for that line, and output values are referenced by indented lines using the variable. There are five basic rule statements: loops (SQL SELECT statements), updates (SQL INSERT and UPDATE statements), if/else branches, identities (SET @X = 1), and IDA-specific functions (e.g., import REDCap instrument data). Additionally, error catching clauses can be added to execute logic if any rule statements fail. Unlike MySQL stored procedures, IDA rules are executed in two steps. First, all database changes output by the IDA rule are written to a separate working database. In the second step, these changes are copied (i.e., committed) from the working database to the target database. The primary advantage of this two-step paradigm is that IDA rules can be developed and tested without changing the target database data, which is preferable to making a copy of the database each time an IDA rule is tested. IDA rules also support temporary tables that provide temporary storage caches while rules are executing and can be used to import CSV content into a rule.

## 5. Data sharing and dissemination

Data sharing is primarily supported by study-designated reviewers who approve or disapprove data access requests submitted from IDA data use applications. Applicants must agree to the terms of data use agreement for each study and must provide information relevant to their proposed use of the study data. Applicants may also be required to submit any manuscripts they have written using study data to the IDA manuscript submission and review subsystem.

Access to data stored in the IDA for a study can be granted in three ways: (1) access to data from one or more institutions in a study can be set in a user management web page, (2) a reviewer can grant guest-level (search and download) access to applicants through semi-automated data application web pages, and (3) all study data can be made publicly accessible to everyone having an IDA user account. Other access levels enable users to upload and download data files acquired at one or more institutions, and data management operations to edit and delete data can be granted.

Downloaders create customized collections of data files from the results of IDA searches and download each collection as a ZIP 64 file. As each archived neuroimaging scan is individually ZIP-compressed and stored with other scans from the same upload using a proprietary IDA “bundle” file format, for each download the requested ZIP-compressed scans are located in their respective bundle files and dynamically assembled into a ZIP 64 stream that is sent to the downloader. This paradigm eliminates the need to create the ZIP 64 file on a local IDA file system before sending and enables support for HTTP range and head requests. The HTTP head request provides the total size of the ZIP 64 file and range options specify a range of bytes to be downloaded from the file. This functionality supports the use of 3rd party download software to establish multiple connections to IDA servers to download different parts of a ZIP 64 file simultaneously, which can decrease the total amount of time needed to download the file. To prevent server overloads, the maximum number of connections allowed for a single user is capped at 10 per IDA server. We have also extended this download paradigm to individual files archived in the IDA, including downloading tables from the IDA database in the comma-separated values (CSV) file format.

In addition to HTTPS requests, which provide a secure data transfer method used by all web browsers, the IDA also supports SSH File Transfer Protocol (SFTP) data transfers. This can be particularly useful for downloaders who are receiving large data files over poor connections. SFTP runs over SSH, has built-in integrity checks, and in our experience guards against file corruption much better than HTTPS, which (beyond TCP) does not incorporate check sum error checking. The IDA SFTP service provides a “virtual” file system using the open-source Apache MINA SSHD library into which users can log in and retrieve files. For security purposes, each download is assigned a random 36-character code that is used as the SFTP login name and expires after 12 h. Downloaders enter their IDA password as the SFTP password and then execute SFTP commands to download files from the virtual directory.

Tabular data is transferred to and from IDA servers with the IDA Sync API, which is a Representational State Transfer (REST) API that can be invoked by standard HTTPS utilities such as CURL and WGET. Authorization keys with limited lifetimes are obtained using IDA user credentials and are used in subsequent REST API endpoints. Responses can be returned in either XML or JSON, and tabular data is downloaded as CSV files. Flexible permissions for users are defined with regular expressions that identify accessible database tables by their names, and additionally filters can be applied to limit data per table row. IDA Sync API endpoints provide functionality for a user to (a) list all accessible tables in a database, (b) list column properties (e.g., data type) of all accessible tables, (c) download data from multiple tables in CSV format, (d) specify search criteria to filter downloaded data, and (e) upload data from a CSV file to an IDA database table. Data may be uploaded as a “partial sync” that updates existing database tables or as a “full sync” that deletes all data not referenced during the update. Additionally, users may define their own NULL characters and date/time formats.

Web applications integrated with the IDA Item API enable files archived in the IDA to be downloaded from web sites external to the IDA. This allows IDA collaborators to design their own web pages with links that access IDA information. The API provides a listing of the IDs, names, descriptions, and versions of all files in study-specific groups defined internally in the IDA. Download permissions for all IDA files accessed by the API endpoints are the same as if the files were directly downloaded from the IDA. External developers first obtain an authorization key for each user by invoking the API with the user’s IDA email address and password. The API provides download links to the latest version of each file as well as older versions. In addition to providing access to files archived in the IDA, tabular data stored in the IDA database may be downloaded as CSV files.

Neuroimaging files archived in the IDA may be downloaded from the IDA using the IDA Java Broker, which can be integrated into external programs written in Java 1.8 or higher. The Broker requires each user’s IDA email address and password and provides a list of all neuroimaging collections created by the user in the IDA. Every neuroimaging scan downloaded by the Broker is transferred as a ZIP 64 compressed stream and is automatically decompressed before being written to the target directory.

## 6. Discussion

**FIGURE 4:**
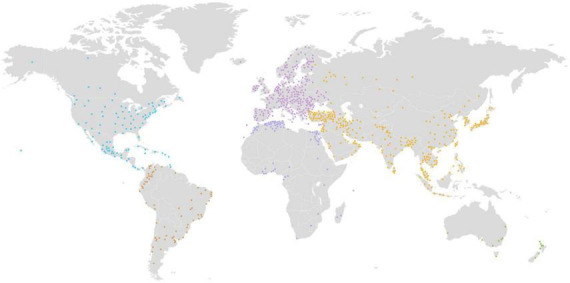
Over 145,000 users from 162 countries have downloaded more than 272 million neuroimaging data scans from the IDA.

To date, the IDA has enabled more than 145,000 users from 162 countries (Figure 4 and Table 2) to download over 272 million neuroimaging data scans. The IDA currently manages 1.5 petabytes of storage, including 79 terabytes of 342 million neuroimaging files. Over 4,900 manuscripts (Weiner et al., 2013, 2015, 2017) have been accepted from IDA studies that require investigators to report their scientific findings. We believe these statistics demonstrate that the IDA functions as an effective repository for data sharing and promotes data reuse.

**TABLE 2:** Top 10 countries downloading neuroimaging files from the IDA in gigabytes (GB).

Country	Downloaded (GB)
United States of America	866,105
China	238,059
Canada	117,341
Korea	110,357
Germany	87,606
United Kingdom of Great Britain and Northern Ireland	74,512
Japan	72,339
Hong Kong	69,609
India	69,257
Australia	54,252

## Data availability statement

The original contributions presented in this study are included in the article/supplementary material, further inquiries can be directed to the corresponding author.

## Author contributions

SN, KC, and AT contributed to writing the text of this manuscript. All authors contributed to the article and approved the submitted version.
